# Supplementary figures and images for: Cost-analysis of real time RT-PCR test performed for COVID-19 diagnosis at India’s national reference laboratory during the early stages of pandemic mitigation
Source: PLoS One. 2023 Jan 11;18(1):e0277867. doi: 10.1371/journal.pone.0277867 (PMC9833513; doi:10.1371/journal.pone.0277867)

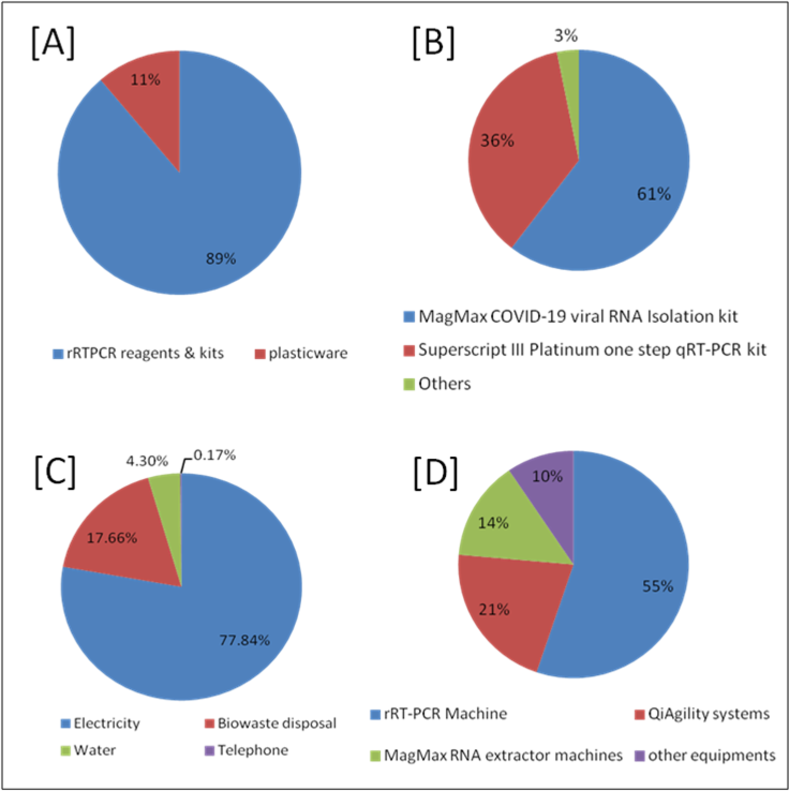

Supplement: S1 Fig — A] Cost distribution among laboratory consumables, B] Cost distribution among rRT-PCR reagents & kits, C] Cost distribution among overhead expenses, D] Cost distribution among laboratory equipments used. (TIF) [file pone.0277867.s001.tif]
