# Supplementary material for: Cost-analysis of real time RT-PCR test performed for COVID-19 diagnosis at India’s national reference laboratory during the early stages of pandemic mitigation
Source: PLoS One. 2023 Jan 11;18(1):e0277867. doi: 10.1371/journal.pone.0277867 (PMC9833513; doi:10.1371/journal.pone.0277867)
Supplement: Attachment — Submitted filename: 01_Oct_Response to Reviewers_2.10pm.docx [file pone.0277867.s003.docx]

**Rebuttal Letter [PONE-D-21-40768]**

We thank the academic editor and the two reviewers for their valuable comments on our manuscript. Our response to each point raised by the academic editor and reviewers is mentioned below. We hope that we have addressed all the points up to the satisfactory level and the manuscript will now be suited for the publication.

Sincerely,

On behalf of all authors,

Dr. Yogesh Gurav

**Academic Editor:**

1. **Please ensure that your manuscript meets PLOS ONE's style requirements, including those for file naming.**

File naming and main body of manuscript was edited in compliance with the style requirements. We hopefully have no disagreement from the style requirements now.

2. **Please provide additional details regarding participant consent. In the ethics statement in the Methods and online submission information, please ensure that you have specified (1) whether consent was informed and (2) what type you obtained (for instance****, written or verbal, and if verbal, how it was documented and witnessed).**

We informed in the ethics statements That the study is being approved by ICMR-National Institute of Virology, Pune Institutional Ethics Committee (No: NIV/IEC/May/2020/D-4). The present cost analysis study does not involve any collection of the clinical specimen by the investigator. The specimens collected at various hospital were sent to the study sites for the diagnosis purpose. The cost of the tests is being analyzed in this study. Hence as per the institute’s ethics committee guidelines, there is no need to have a consent (written or verbal) from the study participant.

3. **PLOS requires an ORCID iD for the corresponding author in Editorial Manager on papers submitted after December 6th, 2016.**

The ORCID iD of corresponding author updated in the Editorial Manager.

4. **Your ethics statement should only appear in the Methods section of your manuscript. If your ethics statement is written in any section besides the Methods, please delete it from any other section.**

The ethics statement was amended in the Methods section only (Page

**Additional Editor Comments:**

1. **Please revise the paper according to the comments of the reviewers. English may be improved considerably although this may not require the use of a native English expert as suggested.**

We revised the manuscript to address all the points raised by the academic editor and both the reviewers. We agreed with the comments in all aspects. We thoroughly reviewed the grammatical errors in the English language and hope that the manuscript is now more understandable, comprehensive, and its conclusions more useful to the public.

**Reviewer #1**

1. **Authors and disclosures section:**

**Financial disclosure: you state that the authors received no specific funding for this work; however if this was done in the course of/as part of their responsibilities work at their respective institutions then this should be acknowledged (i.e. their employers funded the work). I note that you acknowledge the support of HTAIn and ICMR in the acknowledgements section.**

Answer: Financial statement amended in the submission form. This study was not financially supported by any extramural research grant. However, financial support provided by Department of Health Research (HTAIn) in terms of human resource for a HTA resource centre at ICMR-NIV Pune was acknowledged. (Please refer Page 25 line 486-488)

2. **Data availability:**

**Please state where the data is available. Most journals accept that the data is available with the author on request but you do need to state this. Please double check you are following the publication guidelines on this.**

Answer: We have mentioned, “Data will be available on request”.

3. **Authors:**

**Normal practice is to state what role each author took in the analysis (writing, analysis, critical review and contribution to content, design, data collection etcetc).**

Answer: As per journal guidelines, the author’s contributions in terms of conceptualization and supervision, data collection & analysis, writing (Original Draft Preparation), writing (Review & Editing) was already uploaded in the submission form at the time of article submission.

4. **Abstract:**

**Please make sure that the time frame is specified for the unit cost and for the number of tests.**

Answer: We have mentioned in the abstract, the time frame of the study and the number of tests performed. We have used 1 year time frame to estimate the annual unit cost derived from the collected monthly data.( Please refer Page 2, line 31)

5. **Background**
A. **Please clarify your objectives in this section so that the reader knows what you are doing – a lot of your analysis comes as a surprise when you read through the paper. Not only are you looking at the unit cost (as you state) but you are doing so for a peak period in the pandemic; and then you are estimating the average annual cost for PCR testing as well as an estimated average annual unit cost for testing that takes account of peaks and troughs in the pandemic. You also use your data to understand the breakdown of costs across inputs and over time and different levels of testing activity. Please state all these objectives upfront.**

Answer:

The necessary changes are made in abstract as per reviewer’s suggestion.

The monthly cost for rRT-PCR testing as well as an estimated annual average unit cost for testing that takes account of peaks and troughs in the pandemic were investigated. The time frame used to estimate unit cost was one year (July 2020-June 2021). (Page 2, line 29-31)

The data was also used to understand the breakdown of costs across inputs and over time and different levels of testing activity. (Page 2; line 36-37).

B. **The change in unit cost as the pandemic progresses and the correlation between unit cost and outputs is a really important aspect of the study. Please introduce this issue and explain why this is important in the background section.**

Answer: The necessary changes are made in abstract per reviewer’s suggestion.

The unit cost varies over the period of pandemic in proportion with the prices of consumables and inversely proportional with number of tests performed

(Page 3, Line 44-45).

6. **Introduction:**
   A. **Pg1 line 57 – why “like” this test? Isn’t it this test that is gold standard? &Lines56-60 – you state twice the test is gold standard.**

Answer: Yes, the rRT-PCR test is gold standard for detection of SARS_CoV_2 virus. The sentence was rephrased (please refer Page 3 line 58-60) for clarification.

B. **Pg 1 line 64 – please specify that you mean the Indian government.**

Answer: the words ‘Indian government’ is added in the sentence as per reviewer’s suggestion. (please refer Page 3 line 63).

C. **Lines 64-66 - This is not stated in the article referenced. Please access better data on the spend on testing relative to the health budget or explain the important impact that testing has on overall health care expenditure in a different way.**

Answer: Recent reference added on health expenditure for COVID-19 by Ministry of Finance, Govt. of India (please refer Page 4 line 69-71).

D. **Pg2 lines 68-70 – you need to help the reader more here. Please explain what you mean by economic and financial evaluations and efficient resource allocation? (This is not a health economics journal)**

Answer: We added comparative information on economic and financial evaluations to help the reader to understand the basic concept (please refer Page4 line 74-78).

E. **Pg2 lines 72-73 – can you provide some examples of how this has been stated. Also these costs are likely based on prices charged. You need to describe the problem with using charges as an estimate of costs - i.e. they are related to the business model of the provider/lab and have profit margins built in and don't necessarily reflect the actual cost of production. And why do we need to know the cost of production.**

Answer: The necessary correction has made in the sentence as per reviewer’s suggestion. (Please refer Page 4 line 81-85)

F. **Lines 75 – the Gold reference seems out of place – it tells us what gross costing is but doesn’t tell us what method has been used in this instance.**

Answer: The sentence has removed from the manuscript.

G. **Line 79. Start a new sentence after country[12].**

Answer: New sentence started as per reviewer’s suggestion. (please refer page 5 line 89).

**Lines79-82 I don't follow this logic. Are the new labs going to replace state labs? What is the relevance for your costing study? Your rationale is better focused on the lack of data for planning the new laboratories and ensuring adequate investment as well as for use in economic evaluations of different strategies for COVID-19 testing.**

Answer: The existing state labs will not be replaced by newly proposed labs. These can serve as referral laboratories for virological diagnosis in respective regions. The data presented in this study may be beneficial for the resource allocation in the proposed labs.

7. **Methodology:**
A. **Study design and study site**
   i. **Please add a description of the intervention;**

Answer: This is primary costing study, thus no intervention is compared with standard procedure.

ii. **is this lab only costs or is sample taking also included?**

Answer: The cost of the test was calculated once the clinical sample is received at the laboratory. This study does not include the cost of collection of the clinical specimen.

**What are the procedures followed when testing?**

Answer: A brief procedural outline is added in study design section (please refer page 6 line 111-113).

iii. **How long does it take to return a result?**

Answer: The turnaround time for one rRT-PCR test is added in study design section (please refer page 6 line 113-114).

iv. **How do the different cost centres relate to the testing?**

Answer: NIC was the major cost centre in which the actual COVID testing was carried out while AMU was the supportive cost centre included in this costing analysis to estimate the contribution of administration and maintenance units in rRT-PCR testing. (please refer page 6 line 119-121)

v. **Are these dedicated COVID testing sites - what other testing is carried out?**

Answer: NIC was the National Influenza Centre which is the WHO national reference lab for diagnosis of seasonal influenza, H1N1 and other respiratory viruses. During pandemic, this lab was solely working for COVID-19 rRT-PCR based diagnosis for the state of Maharashtra and also a referral lab for the whole country.

vi. **How many tests are carried out per month/year? Please also describe the roles of the different cost centres.**

Answer: We have included the data of tests carried out per month. (please refer page 6 line 114). The role of different cost centers have also included in data collection section. (please refer page 6 line 119-121).

vii. **In addition please state and explain the approach to costing here (activity based costing, bottom up etc)**

Answer: We have mentioned ‘bottom up ‘ approach for costing in study design and study site section (please refer page 5 line 104-107).

B. **Data collection**
i. **Line 100 delete “form the competent authorities”**

Answer: the words “form the competent authorities” has deleted as per reviewer’s suggestion

ii. **Line 103 cost centers – should be cost center (there is only one other)**

Answer: Cost centers is replaced by cost center as per reviewer’s suggestion (please refer page 6 line 119).

iii. **Line 105, line 109, line 112 – please avoid using etc – be specific.**

Answer: word “Etc.” has deleted as per reviewer’s suggestion (please refer to page 6 line 122, 126, 130).

iv. **Line 111 delete “in the proforma… …laboratory” and just state “The data was collected from the stock registers, instrument……**

Answer: the necessary changes has made as per reviewer’s suggestion (please refer to page 6 line 127-128).

v. **Line 113 – What is comprehensive information? What information was collected - i.e. numbers of staff, categories of staff and when they were present?**

Answer: Comprehensive information means all data pertaining to the staff positions and number, job status (regular or contractual or daily wages) and duty timings. It is added in the parentheses for better understanding (please refer to page 7 line 130-131).

vi. **Line 118-9 – please clarify if staff time include both admin and procedure time for each individual staff member?**

Answer: The staff time include procedure time, because in this sentence we are discussing about lab staff only.

**Table 1**

i. **Are all of these costs specific to rRT-PCR testing or are some (e.g. HR) used for other activities as well? If the latter - it would be important to have a column which explains how you allocate these resources to the testing. e.g. how do you know how many of each lab consumable were specifically used for PCR testing?**

Answer: Table 1 specifies the details of cost centers, cost heads, and source of cost data utilized for computing unit cost of RT-PCR test. The allocation criteria used is mentioned in Table 2 for each of the cost heads. Due to pandemic, only RT-PCR testing was done at laboratory. So all the consumables mentioned in both the tables (table 1 and 2) was used only for RT-PCR related work.

ii. **The allocation methods are described partially in different places in the methods section, in table 2, in the results section, and the discussion section. Please bring these all together into one place so that it is clear how these allocations were made and what data sources were used.**

Answer: The allocation methods are described in “Allocation of resources/ apportioning statistics in methodology. (please refer to page 8 line 157).

iii. **Please add a section within the Methodology called “Allocation of resources” or similar; you could then have a separate section on “Price/value data” in which you describe all the price data sources.**

Answer: Section “Allocation of resources/ apportioning statistics:” has added in methodology. (please refer to page 8 line 157). However, we have mentioned the source of the price/value data in table 2. Its is mentioned in the column 4 (Source of data) in Table 2 (Please refer Page 9 line 178).

iv. **Line 153-4 – move the reference to activity based costing and what this is to the study design section**

Answer: The reference was moved to activity based costing (please refer to page 10 line 184-185).

v. **Line 164-165 please make sure it is clear whether this allocation is at the individual or overall level.**

Answer: This is only applicable to the AMU staff at individual level.

vi. **Line 188 is where you start the description of the analysis - Add a section here called “Analysis”. Please add to your “analysis” section a description of how you: calculate early pandemic unit cost; unit cost for each month; total average annual operating cost that accounts for fixed and variable cost; analysis of relationship between service output and unit cost.**

Answer: In contest to both of the above-mentioned comments by the reviewer, instead of mentioning analysis we have mentioned “Unit cost estimation” section to describe early pandemic unit cost for the month of July 2020, total operating cost for one year period (July 2020 to June 2021), and further estimation of unit cost for the consecutive months in a year using the derived annual data that accounted for fixed and variable cost. (please refer to page 11 line 210 and Page 12 line 211-226)

vii. **Also add that you do a detailed descriptive analysis of the breakdown of costs by inputs.**

Answer: The description of analysis of cost breakdown was added in “Unit cost estimation” section as per reviewer’s suggestion. (please refer to page 12 line 229-231)

viii. **Please be consistent in your terminology – sometimes you refer to annual expenditure, sometimes annual operating cost sometimes total cost. Avoid using the word expenditure when doing a cost analysis as this implies you are looking at total value of financial transactions which generally a costing is not trying to do.**

Answer: Instead of ‘expenditure’, we have used ‘cost’ wherever necessary as per reviewer’s suggestion.

ix. **Explain why the fixed and variable cost classification is required i.e. to help estimate the cost variation by month and the average annual cost as they behave differently with respect to different output levels and the nature of the pandemic has led to fluctuations in output.**

Answer: Explanation of the fixed and variable cost classification has been added to “Unit cost estimation” section as per reviewer’s suggestion. . (please refer to page 12 line 217-219)

a. **Table 2**
i. **different types of HR are allocated differently (according to the text) and this is not evident in the table; I suggest you separate the HR into rows categorised according to how the staff time was allocated.**

Answer: HR involved in the study were from two cost centers i.e. NIC and AMU. Although they were on regular, contractual, project, daily wedges, position, the allocation criteria used was time devoted for COVID-19 RT-PCR testing. Only in case of HR from AMU, we have used additional 50% deduction based on personal observation at the time of data collection.

ii. **It is not clear how reference 13 could be used for allocating staff time? Why was this used and not the observational work? Or interviews with staff at the laboratories.
    - I suggest you have a column which states the source of the data (e.g. interview, records etc)**

Answer: As per reviewer’s suggestion instead of ‘reference’, ‘source of data’ is included in last column of table 3 (please refer 259).

iii. **Please explain why you need an assumption and then what the assumption is e.g. for HR at AMU and also explain why this is a reasonable assumption**

Answer: We do agree with the reviewers that there is no need of the assumptions since the investigators themselves have observed the time allocated by the HR at AM. Hence the assumptions worked is being replaced by the terminology i.e., Allocation criteria based on the personal observation’.

iv. **Does indent mean item? Please use an alternative word to indent for an international audience. And please state the source for the consumption data (is this reference 13? If it is reference 13 then please explain in a footnote how this relates to your study.**

Answer: The word ‘indent’ is replaced by ‘Lab records’ as per reviewer’s suggestion. Reference 13 was used to decide what type of allocation criteria we can use in our study. It was not used as a reference for consumption data.

v. **Lab equipment – why was time used here and not number of tests relative to all tests? Did you account for time outside normal working hours?**

Answer: The actual usage of each equipment was used because some lab equipments were used the whole day, while the others were used for limited time during RT-PCR testing. The actual usage of equipment was also usefull in estimation of utility charges e.g. electricity. To get the exact estimation as possible, we have used actual time instead of number of tests.

vi. **Rows 6-9 column 2– so is this based on the information from rows 1 and 2 (i.e. HR)**

Answer: Yes. It is based on the number of HR from rows 1 and 2.

C. **Sensitivity analysis**
I. **Please state which result you are testing in the sensitivity analysis methods section; is it the unit cost or total cost?**

Answer: The sensitivity analysis was carried out for Unit cost data.

II. **Why did you choose these particular variables?**

Answer: During analysis, we found selected variables to be the major cost contributors. Additionally, the prices of consumables were hiked during pandemic, the numbers of samples tested were more, number of HR deputed for COVID-19 testing was more. Hence, we decided to vary these parameters in sensitivity analysis. Also, we thought that these parameters could be important for policy makers to decide resource allocation.

III. **Could you also look at the impact of variations in staff time (or at least the most important staff members) on the overall cost.**

Answer: During pandemic, extra time devoted by the staff was not compensated with extra benefits or perks. Thus, as per our understanding variation in staff time will not have any impact on the cost.

IV. **Can you explain the choice of the 25% range used; and do you apply this to the overall figure or at the level of the individual input?**

Answer: we have used 25% range for sensitivity analysis based on the expert opinion.

V. **You refer to some of the justifications around the choice of variables for the sensitivity analysis in the discussion – this would be better placed here in the methods section.**

Answer: Justification moved to methods as per reviewer’s suggestion. (Please refer Page 13, Line 236-239)

8. **Results**
i. **Line 216 – is annual operating cost what you refer to as expenditure in the methods?**

Answer: This is total cost for conducting RT-PCRT test in reference period (July 2020 to June 2021). We have also replaced ‘expenditure’ by ‘cost’.

ii. **Table 3 please add the timeframe to the title and indicate which costs are reported in the table.**

Answer: The time frame and type of costs are added in title of table 3 as per reviewer’s suggestion. (please refer to page 14, line 259-260)

iii. **Table 3 - For fund distribution column – is this percentage of unit cost or total cost? I am assuming that the unit cost is the peak month cost and the total cost is the extrapolated cost and therefore the % breakdown would be different. But this is also not clear and needs to be labelled in the table.**

Answer: The percent distribution specifies for unit cost as well as for total cost based on annual data. It is not for the peak month data.

iv. **Table 3 - Tidy up table. No need for decimal places; use comma after each three digits, total cost might be better presented in thousands. Align numbers to right of column for numbers; left of column for text.**

Answer: All necessary corrections has made in the table 3 as per reviewer’s suggestions.

v. **Table 3 - For the cost breakdown, could you add sub rows for the important cost elements to this table and then eliminate the need for the figure 1 (or figure 1 can go in appendix)**

Answer: The sub rows are included in table 3 and we have given figure 1 in supplementary material as per reviewer’s suggestion.

vi. **The recurrent cost analysis is not referred to in the methods section -please do so.**

Answer: The recurrent and capital cost analysis are included in methods under analysis section as per reviewer’s suggestion. (please refer to page 11 line 207-209)

vii. **Please extract all references to methodology from the results and ensure that these are documented in the methods section and avoid duplication e.g lines 230-231; 258-9; 269-70**

Answer: lines 230-231; 258-9; 269-70 are moved to methodology section as per reviewer’s suggestion.

viii. **Table 4 - what is the cost contribution of the different types of staff? can you add this? It would be an interesting addition; are the costs here monthly or annual (and/or relating to peak pandemic)**

Answer: We have added cost distribution of the different types of staff in table 4. These cost are annual cost in peak of pandemic.

ix. **Figure 2 could be tidier…..how about using stacked bar charts? Or use a legend rather than all the label and lines?**

Answer: The purpose of the figure 2 is to show the percentage of variable cost and fixed cost and in variable cost how much is the percentage and lab and non-lab consumables. We thought that the Pie charts would be suitable to shows more explanation for this data than stacked bar charts. Thus, we would like to retain the Pie charts along with all labels which helps to conveys adequate/right information.

x. **Figure 3 – the axes are a bit off; you should be able to cut them at a point >0 so you don’t have such a big gap. The legend needs more detail.**

Answer: The necessary changes has made in the Figure 3 as per reviewer’s suggestion. Now this figure is mentioned as figure 2.

xi. **Line 293 -295 – a percentage figure is reported but its not clear what this represents – does this relate to the percentage difference resulting from the sensitivity analysis?**

Answer: We would like to inform reviewer that percent figures (19%, 2.1%, 0.38 %, 1.8 % and 0.57%) mentioned in the previous draft (page no 293 to 295) were not related to the percentage difference resulting from the sensitivity analysis. These percentages reflect the variation in the unit cost of the RT PCR test when the parameter (price of RT PCR reagents and Kits among total consumables) altered by 25% increase or 25 % decrease in the prizes. It informs that by 25% increase or 25 % decrease in the prizes of RT PCR kits and reagents, there is change in unit of the RT PCR test by 19% . Similarly there will be change in unit cost of the RT PCR test by other parameters as mentioned in line (293-295 in previous manuscript). (please refer to page 18 line 322-329) The sentences has rewritten in manuscript for more clarification.

xii. **Line 296 – What do you mean by does not change much? Is this relative to something?**

Answer: The unit cost dosen’t change much when discount rate is varied as compared to other parameters variation.

xiii. **Line 298-99 – why is this regression not mentioned before?**

Answer: The regression analysis is introduced in methods as per reviewer’s suggestion (please refer to page 12 line 231-233).

xiv. **Line 300-301 – please explain either in the methods or in the discussion why this negative correlation is so important particularly in a situation with fluctuations in demand.**

Answer: As per the reviewers suggestion we have incorporated following explanation in discussion “This negative correlation indirectly reveals the relation between the unit cost of RT PCR test and optimal resource utilization. The policy maker could easily observe the optimal utilization of the resources by following this negative correlation.” (please refer to page 24 line 461-464)

9. **Discussion**
1) **Line 317 – Which rising costs? Its not clear what you are referring to here.**

Answer: The rising cost of RT-PCR test in pandemic situation (please refer to page 19 line 350-351)

2) **Line 329 - how have you calculated salary? did you include benefits? which is important for comparison with contract personnel.**

Answer: The HR salary was calculated as reported by individual HR and confirmed through the pay slip record issued by the accounts section of the institute. No extra benefits were given for COVID-19 duty to any staff. However, difference in salary structures of permanent & contract staff is justified and mentioned. (please refer to page 19 line 357-361)

3) **Line 332-3 – in what context was the pay lowered to half – in practice or for the analysis? And why?**

Answer: The pay of AMU staff was lowered to half during analysis and on the basis of time allocation for COVID-19 rRT-PCR based testing only (also mentioned in Table 2). The basis of pay lowered as half (salary reduction) was based on personal observation by the investigator during data collection procedure. Thus only 50% salary of AMU staff was considered during the analysis so as to estimate the per Unit cost of RT PCR Test. (please refer to page 19 line 359-360)

4) **Line 336 – you need to justify why overall hospital costs and their structure are relevant here when the costing topic here is related to diagnostics and laboratories which will necessarily be very different (e.g. there is no patient care). The only point of relevance is that on wage structures.**

Answer: We feel that the principle of costing for the various procedures/services in the hospitals and costing methods for calculating unit cost of laboratory test in a hospital/laboratory are the same. The necessary justification for the relevance of hospital costs and their structure is being added in the manuscript. (please refer to page 20 line 384-385)

5) **Line 340-343 – hospital cost structures are not relevant to your analysis; they provide completely different services so please remove this sentence. Please find some diagnostics costings and then make the comparisons**.

Answer: We incorporated some recent references as per reviewers suggestion. In Imani et al 2018 and Muniyandi et al 2021, they have shown HR contribution is highest in unit cost of diagnostic test (please refer to page 20 line 370-373). Moreover, we have also compared it with hospital cost structures as we thought that diagnostic service is one among the various healthcare services provided by hospitals, hence tried to make comparison

6) **Line 352-3 – its an important point that you did not include the additional hours of staff during the pandemic. Please can you add a scenario to your sensitivity analysis that accounts for this. I believe your unit cost will rise for the peak pandemic periods and bring it more in line with the months of low testing rates.**

Answer: We agree to reviewers believe of rising unit cost after including additional hours of staff devoted in pandemic situation. To perform sensitivity analysis of the staff hours, we require a basic data of the additional monitory benefits given for an hour of additional service provided. Unfortunately, we do not have such data. Hence, we have addressed this issue by performing sensitivity analysis of HR salary.

7) **Pg 17-21 – there is one long paragraph – this is too long. Please split this into separate paragraphs that make separate points. Also don't repeat methodology – this should all be in the methods section. There's too much detail here that should either be in the methodology or in a supplement. When you’re identifying the key limitations, state why its a problem and what you've done to mitigate this e.g. sensitivity analysis and whether its an important bias or not. If there are multiple limitations you may need more than 1 paragraph.**

Answer: Separate Paragraphs added to discuss separate points as per the reviewer’s suggestion.

8) **Pg line 397-8 On what basis is this cost-effective? How does being used to its maximum potential imply cost-effectiveness?**

Answer: During analysis and annualization of capital items, we have apportioned the total cost of equipments based on actual usage (in hrs) per day. During pandemic, all the automated equipments were used to its maximum potential hence seems cost-effective during sample overload. In contrast, if sample load would be less, the usage of automated equipments would be less and the costlier equipments will not be utilized to its maximum potential, hence not seems cost effective.

9) **Line 405 – The alternative possibility for what? Please explain.**

Answer: We have used activity based apportioning while computing infrastructure cost, that’s why the infrastructure cost was almost negligible to the overall cost. The line was reframed and added in the text. (please refer to page 21 line 399-400)

10) **Line 407 – without the context of what the AMU function is, its difficult to understand whether this is important or not? Please explain under the study setting section.**

Answer: AMU is the supportive (minor) cost center which we have included in this study while the major cost center was NIC. Explanation added under study design & setting section of methodology. (please refer to page 6 line 109-111)

11) **Line 409 (and elsewhere) – write percentage out in full**

Answer: we have added ‘percentage’ in full form in all manuscript as per reviewer’s suggestion.

12) **Line 411-413; line 422-23 – this is all methods and belongs in the methodology**

Answer: Content moved to sensitivity analysis section under methodology as per reviewer’s suggestion. (Page 13 Line 240-243; Page 5 line 104-107; page 6 line 108-109)

13) **Line 426-7 – so do you mean that these items have been excluded? What are the implications of this?**

Answer: Yes, these supportive services were excluded from the study design. Inclusion of all these may reflect the better picture of indirect costs associated with rRT-PCR based COVID-19 diagnosis.

14) **line 429 -431 – the study does more than this – it also explores how costs change with the fluctuations in demand and provides an understanding of the cost drivers for PRC testing.
    Overall the discussion is very much focussed on the limitations and methodological weaknesses – can you add something on the policy implications of the findings – particularly from figure 4 and the HR issues (e.g. is there excess capacity to ensure the testing is carried out or are staff working over time and not been recompensed)**

Answer: The inverse relationship between unit cost and number of sample tested, point’s outs capacity of system to have maximum benefit in optimum resources. The regression analysis in our study gives the range of unit costs which helps policy maker or program manager to monitor optimal use of resources. We tried to mention the limitation of this study and informed that how cost changes with the fluctuations in demand. In upcoming months more zonal National Institute of Virology (Central zone, south Zone, East zone and north zone) are upcoming in India. Such costing study could help in estimating the unit Cost of the test in such labs and possible clinical samples flow to the laboratory during the public health emergency. India is having high heterogeneity among the various states and state specific data are needed in future.

**Typos:
Pg1 Line 50 – add “the” after pandemic.**

Reply: We have added The after the word pandemic (Page 3, Line 52)

**Pg1 line 54 – what do you mean by suspect?**

Reply: We have mentioned it as suspect case of COVID-19 (Page 3 line 56). A suspect case means a case with specific symptoms compatible to the disease under investigation.

**Pg1 line62 – delete “being”**

Reply: The word being is deleted.

**Pg2 line 78 – add “The” before Government**

“the” added before the Government (Page 5 line 88)

**Reviewer #2:**

**Points to note:**

1. **The cost of training the personnel who were both contractual and full time from other sections, and were used to perform highly technically exacting molecular tests, has not been analysed.**

Answer: All HR who were already trained in rRT-PCR testing and doing molecular testing in their respective sections were deployed in COVID lab. They used the SOP of the tests. Although no extra benefits were paid to the deployed staff for COVID duties, hence not included in the study.

2. **The following statement needs to be justified for setting up similar laboratories throughout the whole country as suggested by the authors "Our study estimates can be used to ascertain the cost effectiveness of free of cost provisioning of this service by the government and can also be used for setting up a similar level of lab infrastructure throughout the country".
   The Covid pandemic saw all Govt. tertiary care and headquarter hospitals set up molecular laboratories to carry out Real Time RT PCR tests and were performing large number of tests with existing manpower. So workable laboratories have already been set up.**

Answer: The last sentence mentioned in the abstract informs that the per unit cost of the COVID-19 rRT-PCR test may help in determining the cost effectiveness of the intervention where the test results were provided free of cost to the consumers through the Government. The government could also know the cost of COVID-19 rRT-PCR they got (government perspective) while providing the lab diagnosis. The costing analysis will help the Government to know how much money they are spending as most of the time they provide services in subsidized rate to the people. It will further help them in budget allocation. This costing exercise would help to understand the value for money they have invested for the lab diagnosis of COVID-19 test. Our study would help the Government to understand the value for money they invested for laboratory diagnosis of COVID-19, budget allocation, integration and decentralization of laboratory services so as to help for achieving universal health coverage. We have modified the sentence keeping in mind the above justification (Please refer Page 3 line 43-48) .

3. **The Central Govt. Health and Research Department (ICMR) has already set up several Virus research Diagnostic laboratories across the country. The authors could advocate cost analysis keeping in mind the already existing VRDLs.
   Do the authors advocate more such laboratories to the level of the National Reference Lab ?**

Answer: We do agree that Central Govt. Health and Research Department (ICMR) has already set up several Virus research Diagnostic laboratories across the country and most of these laboratories are in the various Medical Colleges in various states in India. In the initial phase of the pandemic, ICMR-NIV has provided them all necessary reagents and support. As per the ambitious project of the Govt. of India Atmanirbhar Bharat (Self-sufficient India), Govt is in process of having four Zonal National Institute of Virology (NIV) institutes (under the main NIV at ICMR-NIV, Pune) in upcoming years. We do believe that this study would help the Government to understand the value for money they invested to expand the laboratory network. Further integration and decentralization of laboratory services by the Government would help to reach maximum people so as to help in achieving the universal health coverage.
